# Breast cancer hormone receptor levels and benefit from adjuvant tamoxifen in a randomized trial with long-term follow-up

**DOI:** 10.2340/1651-226X.2024.40493

**Published:** 2024-07-05

**Authors:** Helena Fohlin, Anna Nordenskjöld, Johan Rosell, Mårten Fernö, Tommy Fornander, Lisa Rydén, Lambert Skoog, Bo Nordenskjöld, Olle Stål

**Affiliations:** a Regional Cancer Center South-East Sweden and Department of Biomedical and Clinical Sciences, Linköping University, Linköping, Sweden; b Department of Oncology, Institute of Clinical Sciences, Sahlgrenska Academy, Sahlgrenska University Hospital, Gothenburg Sweden; c Division of Oncology, Department of Clinical Sciences Lund, Lund University, Lund, Sweden; d Department of Oncology and Pathology, Karolinska Institute, Stockholm, Sweden; e Department of Clinical Sciences Lund, Division of Surgery, Lund University, Lund, Sweden; f Department of Biomedical and Clinical Sciences, Linköping University, Linköping, Sweden

**Keywords:** Breast cancer, estrogen receptor, tamoxifen, long term

## Abstract

**Background:**

Hormone receptor positivity predicts benefit from endocrine therapy but the knowledge about the long-term survival of patients with different tumor receptor levels is limited. In this study, we describe the 25 years outcome of tamoxifen (TAM) treated patients.

**Patients and methods:**

Between 1983 and 1992, a total of 4,610 postmenopausal patients with early-stage breast cancer were randomized to receive totally 2 or 5 years of TAM therapy. After 2 years, 4,124 were alive and free of breast cancer recurrence. Among these, 2,481 had demonstrated estrogen receptor positive (ER+) disease. From 1988, the Abbot enzyme immunoassay became available and provided quantitative receptor levels for 1,210 patients, for which our analyses were done.

**Results:**

After 5 years of follow-up, when all TAM treatment was finished, until 15 years of follow-up, breast cancer mortality for patients with ER+ disease was significantly reduced in the 5-year group as compared with the 2-year group (hazard ratios [HR] 0.67, 95% confidence intervals [CI] 0.55–0.83, *p* < 0.001). After 15 years, the difference between the groups remained but did not increase further. A substantial benefit from prolonged TAM therapy was only observed for the subgroup of patients with ER levels below the median (HR = 0.62, 95% CI 0.46–0.84, *p* = 0.002). Similarly, patients with progesterone receptor negative (PR-) disease did benefit from prolonged TAM treatment. For patients with progesterone receptor positive (PR+) disease, there was no statistically significant benefit from more than 2 years of TAM.

**Interpretation:**

As compared with 2 years of adjuvant TAM, 5 years significantly prolonged breast cancer-specific survival. The benefit from prolonged TAM therapy was statistically significant for patients with ER levels below median or PR-negative disease. There was no evident benefit from prolonged TAM for patients with high ER levels or with PR+ tumors.

## Introduction

During the last 40 years, tamoxifen (TAM) has been available as adjuvant therapy for breast cancer (BC) patients. It remains a first-hand choice for premenopausal patients. After the publication of the Swedish Breast Cancer Group comparison of 2 and 5 years of TAM therapy, 5 years became standard [1]. Patients benefit from TAM therapy even several years after the therapy has been finished. The duration of this carryover effect is not well established, but Ekholm et al. estimated it to last for at least 15 years for premenopausal patients [2]. The benefit from TAM is restricted to patients with estrogen receptor positive (ER+) disease, but the long-term predictive value of quantitative ER and PR measurement requires further studies. In the EBCTCG overview from 2011, neither ER levels nor PR in combination with ER+ status provided independent predictive information [3]. In contrast, when we analyzed the Stockholm TAM trial, we observed a significantly prolonged recurrence-free survival for patients with disease positive for both ER and PR as compared to those with tumors positive for ER only [4]. Here we study the influence of hormone receptor levels on long-term outcome for patients participating in a randomized comparison between 2 and 5 years of adjuvant TAM therapy. In this study, quantitative receptor levels for ER and PR were determined with Abbot enzyme immunoassay (EIA), but nowadays quantitative values may be assessed from mRNA.

## Patients and methods

**Figure 1:**
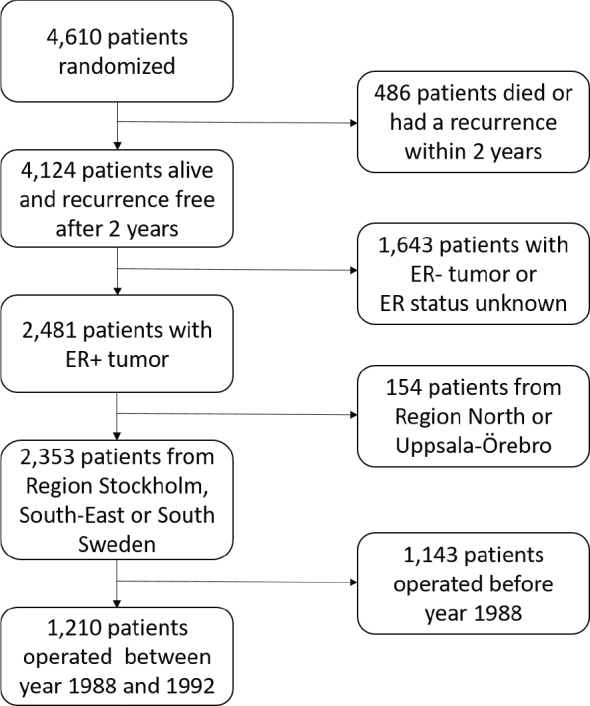
Consort diagram.

The comparison between 2 and 5 years of adjuvant TAM was planned and organized by the Swedish Breast Cancer Group and involved five regional BC study organizations. During the period of 1983–1992, a total of 4,610 postmenopausal women younger than 75 years with early stage invasive BC were entered into a randomized trial comparing 5 and 2 years of TAM. The randomization was done separately for each study center. Two years after surgery, 4,124 women remained alive, had no recurrence, and no contralateral BC. Among these, 2,481 had demonstrated ER+ disease (Figure 1).

Before 1988, ER levels were determined with isoelectric focusing of ER in tumor cytosols [1]. From 1988, when EIA based on ER and PR-specific antibodies were available, this was used by the study organizations in South Sweden, South-East Sweden and Region Stockholm [5]. These study centers provided data on EIA levels for 1,210 patients distributed as follows: 481 patients with Stage II disease from South Sweden, 260 patients with Stage I and 317 patients with Stage II-IIIA from South-East Sweden and 152 patients with Stage I-IIIA from Region Stockholm.

The 1,210 patients were divided into two groups, defined as ER high and ER low, representing ER levels above and below the median value obtained in each study center.

Data on survival and causes of death were obtained from the National Board of Health and Welfare.

### Statistical analyses

The cumulative proportion of BC mortality was estimated using the Kaplan-Meier method. Time for follow-up was defined as the time from randomization until death or last observation (December 31, 2019). Data on date of death and cause of death was available until year 2019. Patients were censored at last follow‑up or death due to other causes than BC.

Hazard ratios (HR) and 95% confidence intervals (CI) were estimated using the Cox’s proportional hazards model stratified by trial center, and the p values were obtained from two-sided Wald tests. Analyses were done by the intention-to-treat.

A *p* value of < 0.05 was considered to be statistically significant. The statistical analyses were performed using STATA/SE 13.1 [6].

## Results

**Figure 2:**
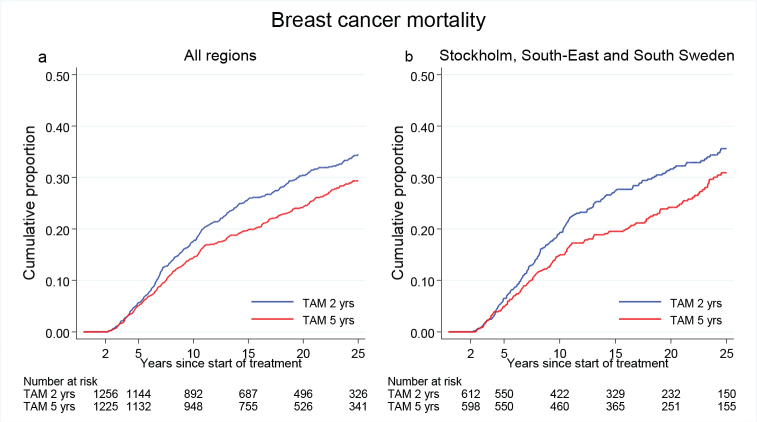
(a) Breast cancer mortality in the treatment group of 5 years TAM compared with 2 years for patients with ER+ breast cancer undergoing surgery between 1983 and 1992. Including all regions. (b) Breast cancer mortality in the treatment group of 5 years TAM compared with 2 years for patients with ER+ breast cancer undergoing surgery between 1988 and 1992. Including Region Stockholm, South-East and South Sweden.

Figure 2a illustrates BC mortality for all patients diagnosed between year 1983 and 1992 with ER+ disease. During the first 15 years after surgery, BC mortality was significantly reduced in the 5-year group. Beyond 15 years of follow-up, the difference between the groups remained, but did not increase further (Table 1). From January 1, 1988, ER and PR were analyzed with EIA. For patients operated after this date, the BC mortality was similar to that in the entire population with ER + disease (Figure 2, Table 1).

**Table 1:** Breast cancer mortality in the treatment group of 5 years TAM compared with 2 years for patients with ER+ breast cancer undergoing surgery between 1983 and 1992^a^, and between 1988 and 1992^b^

Years after surgery	Number of events		HR 5 versus 2 years	95% CI	*p*
**1983–1992**	TAM 2 years (n = 1,256)	TAM 5 years (n = 1,225)			
>2	361	303	0.77	0.66–0.90	0.001
2–5	70	61	0.85	0.60–1.20	0.35
5–15	222	164	0.67	0.55–0.83	<0.001
15–	69	78	1.01	0.73–1.39	0.97
**1988–1992**	TAM 2 years (n = 612)	TAM 5 years (n = 598)			
> 2	180	150	0.76	0.61–0.94	0.01
2–5	39	29	0.73	0.45–1.18	0.19
5–15	112	79	0.63	0.47–0.84	0.002
15–	29	42	1.26	0.79–2.03	0.33

a Including all regions.

b Including Region Stockholm, South-East and South Sweden.

CI: confidence intervals; HR: hazard ratios.

**Figure 3:**
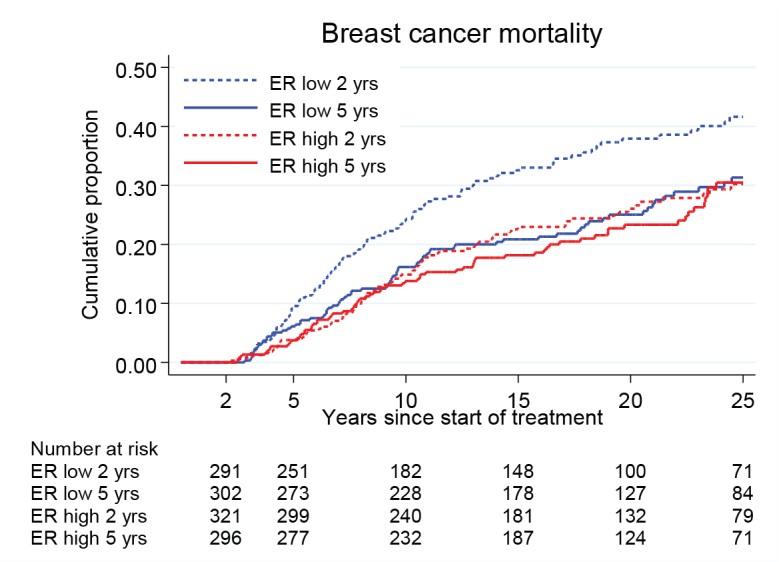
Breast cancer mortality in the treatment group of 5 years TAM compared with 2 years for patients with ER+ breast cancer undergoing surgery between 1988 and 1992 and stratified by ER values above and below the median (ER high and ER low). Including Region Stockholm, South-East and South Sweden.

Figure 3 and Table 2 illustrate the BC mortality for patients with ER positivity divided into two groups, defined as ER high and ER low, representing ER levels above and below the median value, as measured with EIA. In the ER high group, the BC mortality was similar in both groups, independently of TAM treatment duration. In contrast, in the ER low group, the BC mortality was significantly reduced in the treatment group of 5 years TAM. For these patients, the BC mortality rate was similar to that observed for patients in the ER high group. There was a nearly statistically significant interaction between ER level and TAM treatment duration (*p* = 0.06).

**Table 2:** Breast cancer mortality in the treatment group of 5 years TAM compared with 2 years for patients with ER+ breast cancer with ER value above the median (ER high and ER low). Including patients undergoing surgery between 1988 and 1992 in Region Stockholm, South-East or South Sweden.

Years after surgery	Number of events		HR 5 versus 2 years	95% CI	*p*
**ER high**	TAM 2 years (*n* = 321)	TAM 5 years (*n* = 296)			
>2	80	72	0.92	0.67–1.26	0.61
2–5	12	11	1.01	0.45–2.29	0.98
5–15	54	39	0.75	0.50–1.13	0.17
15–	14	22	1.48	0.75–2.90	0.26
**ER low**	TAM 2 years (*n* = 291)	TAM 5 years (*n* = 302)			
>2	100	78	0.62	0.46–0.84	0.002
2–5	27	18	0.60	0.33–1.09	0.094
5–15	58	40	0.53	0.35–0.79	0.002
15–	15	20	1.04	0.53–2.05	0.90

CI: confidence intervals; HR: hazard ratios.

**Figure 4:**
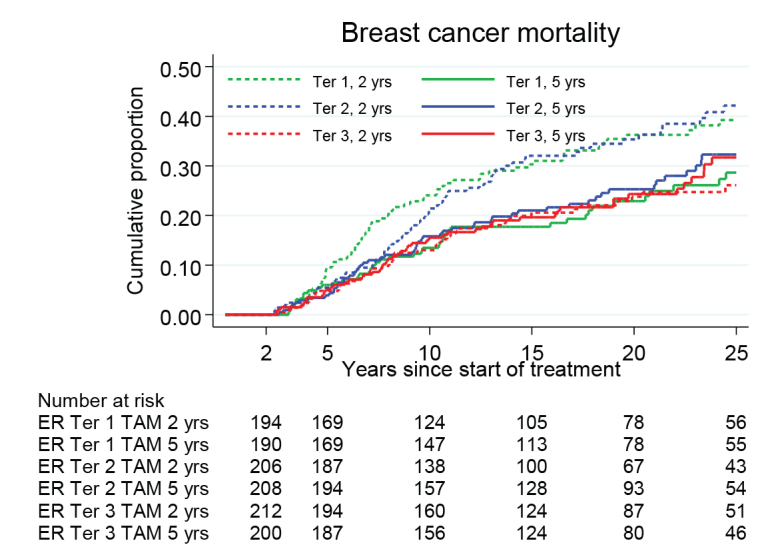
Breast cancer mortality in the treatment group of 5 years TAM compared with 2 years for patients with ER+ breast cancer undergoing surgery between 1988 and 1992 and stratified by ER values in tertiles. Including Region Stockholm, South-East and South Sweden.

By further stratification of the patients into three groups based on the tertiles of ER+ levels, there was a trend that patients with higher values of ER had less benefit from prolonged TAM. The HR values for 5 versus 2 years of TAM were 0.61 (95% CI 0.42–0.90, *p* = 0.012) for the tertile of patients with the lowest tumor ER levels, 0.64 (95% CI 0.45–0.92, *p* = 0.016) for the middle tertile and 1.09 (95% CI 0.73–1.63, *p* = 0.67) for the tertile with the highest ER levels. The interaction between ER and TAM had a p value of 0.03 over the whole follow-up period and 0.06 between 5 and 15 years as illustrated in Figure 4.

**Figure 5:**
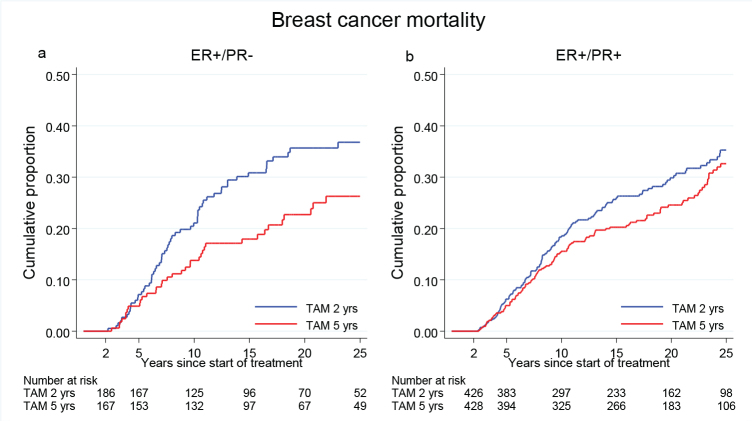
Breast cancer mortality in the treatment group of 5 years TAM compared with 2 years for patients with ER+ breast cancer undergoing surgery between 1988 and 1992 and stratified by PR status. Including Region Stockholm, South-East and South Sweden.

Patients with ER +/PR- disease treated with TAM for 5 years had a significantly reduced BC mortality as compared with those treated for 2 years only (HR = 0.64, 95% CI 0.43–0.97, *p* = 0.03). In contrast, for patients with ER+/PR+ disease there was no statistically significant difference in breast cancer mortality between the treatment groups (HR = 0.82, 95% CI 0.63–1.06, *p* = 0.12) (Figure 5). However, the interaction between PR and TAM was not statistically significant (*p* = 0.25).

## Discussion

More than two decades ago, cytosol EIA using hormone receptor-specific antibodies was replaced by immunohistochemistry (IHC). This was an important progress in the management of BC, making it possible to identify ER and PR in tumor cells from formalin fixed paraffin-embedded tumors. The proportion of tumor cells identified as receptor positive is generally reported from the pathology, but it is debated which cut-off motivates adjuvant endocrine therapy, mostly either 1% or 10%. Lindstrom et al. have shown that heterogeneity in ER staining is associated with reduced benefit from adjuvant TAM [7]. IHC can be considered as a semi-quantitative method. In the review by Noordhoek et al. [8] of the literature on outcome correlated to levels of ER and PR quantitatively measured with IHC, they found no clear evidence for using IHC levels of ER and PR, neither as prognostic nor as predictive markers. They recommended using a qualitative IHC status in treatment considerations. Viale et al. [9], Bartlett et al. [10] and Dowsett et al. [11] analyzing the BIG 98, TEAM and ATAC trials found that IHC based levels of ER and PR did not identify patients with differential relative benefit from aromatase inhibitors over TAM but reported that the receptors provided prognostic information within each treatment group.

In the NSABP B14 trial Fisher et al. [12] compared 5 years of adjuvant TAM with placebo and used sucrose density gradients or dextran-coated-charcoal techniques for ER quantitation. ER levels above 50 fmoles per mg protein tended to indicate improved disease-free survival for TAM treated patients (*p* = 0.07). Furthermore, findings from the EBCTCG overview [13] in 1998 showed a greater proportional reduction in BC recurrence and mortality in women who had high ER concentrations (at least 100 fmol per mg cytosol protein) in the tumor compared with women who had tumors with lower ER.

In our study, we used quantitative values of ER and PR as measured with EIA to evaluate the benefit from prolonged TAM treatment. In the future, quantitative ER and PR mRNA may be assessed. One example of a gene expression-based signature evaluating estrogen signaling is the Breast Cancer Index (HOXB13/IL17BR). The aTTom trial reported a correlation between a high level of BC index and benefit from prolonged endocrine therapy [14, 15]. The Breast Cancer Index showed a weak negative correlation with ER and PR, and although the authors could not show statistical significance, the trend indicated more benefit from the endocrine treatment for decreasing ER mRNA as TAM was prolonged from 5 to 10 years.

Already in an early report from the comparison of 2 and 5 years of TAM, Ferno et al. [16] found that the benefit from 5 years of therapy was statistically significant for patients with less than median tumor levels of ER but less pronounced for patients with high levels.

In this study, we confirm that patients with less than median tumor ER levels benefit from prolonged TAM therapy. It is not reasonable that EIA should be reintroduced in the management of BC, but quantitative levels of ER and PR may be assessed by other modern methods, such as mRNA gene expression [17]. Previously, we have shown that PR mRNA positivity may be used to predict adjuvant TAM benefit [18].

Five years of adjuvant TAM do not fit all patients although previous studies showed that the risk for contralateral BC and lung cancer decreased with prolonged TAM while the risk for endometrial cancer increased, and the incidence for all types of cancer was similar between the treatment groups [19, 20]. In our study, there was no evident benefit from prolonged TAM treatment of the tertile of patients with the highest tumor ER levels. If the results are confirmed by other studies, patients having a low risk of recurrence and tumors strongly positive for ER and PR may be informed that 2 years of TAM therapy is a reasonable duration. Some patients have severe side effects during endocrine treatment [21] and discontinue adjuvant therapy. Clinicians may encourage those with high tumor ER levels to endure 2 years of adjuvant therapy despite side effects.

Patients with low tumor ER levels and patients with ER+/PR- disease had a prolonged breast cancer-specific survival after 5 years of TAM therapy as compared to after 2 years and should be encouraged to continue the therapy for 5 years.

In the S:t Gallen Guidelines, it is stated that almost all patients with ER+ disease are candidates for adjuvant endocrine therapy and that for high-risk tumors aromatase inhibitors should be considered. Also, for many patients, endocrine therapy should be extended to more than 5 years [22].

One strength of our study is that almost all patients that fulfilled the inclusion criteria during the study period were included and randomized to either 2 or 5 years of TAM. A limitation is that only tumors from patients diagnosed in 1988 or later were analyzed with EIA, which is a more reliable method for quantification of ER and PR than ligand-based techniques. For this reason, we excluded the patients diagnosed before 1988 so that approximately half of the patients with demonstrated ER+ disease remained for the statistical analyses. Another limitation may be that the ER and PR measurements were determined in different laboratories. However, it has previously been shown that there was excellent concordance between the laboratories [5].

Taken together with our previous finding that patients with breast cancer positive for both receptors had prolonged benefit from 2 years of TAM compared with patients having tumors positive for ER only, our data show that PR positivity tends to increase breast cancer sensitivity to adjuvant TAM [4].

TAM has a carryover effect, reducing the risk of breast cancer mortality for a long time after the treatment has been finished. The carryover effect is commonly defined as a continued reduction of breast cancer-related events after cessation of TAM treatment. The mechanisms of this phenomenon are unknown but have been observed in other TAM trials with long-term follow-up [23, 24]. The effect was demonstrated to last for at least 15 years in patients having only 2 years of TAM by Ekholm et al. [25]. In the present study, we also observed prolonged breast cancer survival for patients treated for 5 years TAM compared with 2 years. The difference between the groups increased up to 15 years after surgery and remained thereafter. Since we have no untreated control group, we cannot estimate the duration of the carryover effect. However, our data suggest that for patients with high tumor receptor levels, 2 years of TAM also provides a clinically relevant carryover effect.

## Data Availability

The data will not be shared as the study participants did not consent to sharing their data in a public repository.

## Highlights

- As compared with 2 years of adjuvant TAM, 5 years significantly prolonged breast cancer-specific survival.
- The benefit from prolonged TAM therapy was statistically significant for patients with tumor ER levels below median or PR negative disease. There was no evident benefit for patients with high tumor ER levels or with PR positive disease.
- Our data suggest that for patients with high tumor receptor levels, 2 years of TAM provides a long carryover effect.
